# Cold war of strains: the ‘Bulgarian’ BCG vaccine between Paris, Copenhagen, and Moscow (1940s–1950s)

**DOI:** 10.1017/mdh.2025.10044

**Published:** 2025-10

**Authors:** Milena Angelova

**Affiliations:** Anthropology, New Bulgarian University, Sofia, Bulgaria

**Keywords:** Tuberculosis, BCG, Bulgaria, Sovietisation, ‘Semashko’ healthcare system, Communist regime, Srebra Rodopska

## Abstract

This article deals with the tuberculosis policy in Communist Bulgaria from the 1940s to the end of the 1950s. The focus is on the BCG vaccination as the major preventive tool. The article’s reconstruction of decision-making draws on evidence from archive records produced by the Bulgarian Ministry of Health. The main question guiding the research is how past Bulgarian experiences on the one hand, and international traditions, on the other, influence medical opinion and state policy towards tuberculosis and patients with tuberculosis. How did the Cold War context shape BCG vaccination policy? The author presents the story of the ‘Bulgarian’ BCG strain, which was made possible by the international research networks and travels of the Bulgarian scientist Srebra Rodopska (1913–2006). Her story has recently been rediscovered and made popular in Bulgaria, in the context of debates about COVID-19. This article aims to correct the public history narrative, which has thus emerged by placing the story of the BCG vaccine within its Cold War context. The author pays attention to dependencies between medicine and politics, and to the role of the state. Despite the popular story of Rodopska as the inventor of a ‘Bulgarian’ BCG strain and vaccine, what actually happened was that in Bulgaria of the 1950s and 1960s, the Soviet strain and vaccine production technique were used. This was also due to Soviet pressure to follow the Soviet model of public health infrastructure.

‘Tuberculosis can only be defeated in a socialist state.’^1^

– recommended slogan for the anti-tuberculosis dispensary in Bulgaria, 1950s

## Introduction

During the 2020 COVID-19 pandemic and the search for treatments and vaccines, a scientific and media hypothesis became popular, suggesting that BCG may also offer some protection against the global viral threat.^2^ In a study, Richard Bluhm and Maxim Pinkovskiy specifically analysed the incidence of coronavirus cases along the former East–West German border, using modern econometric techniques, to examine whether historical differences in vaccination policies could account for different infection rates in the former German Democratic Republic (GDR) and the Federal Republic of Germany (FRG).^3^

In Bulgarian media and social networks, in March to April of 2020, opinions also began to circulate that the country’s mandatory BCG vaccination against tuberculosis – or the administration of the vaccine since 1951 – could have a potential immunostimulating effect against COVID-19. In these publications, the name of Dr. Srebra Rodopska also appeared, as the ‘discoverer of the Bulgarian BCG vaccine’ from the late 1940s and early 1950s.^4^ Literally, within days, the biography of the until-then-forgotten bacteriologist became known to hundreds of thousands of people in Bulgaria, and within a few weeks, Dr. Rodopska became an almost legendary figure. Presenting the case of Dr. Srebra Rhodopska, the article examines both the broader contexts and the interdependencies between medicine, politics, and ideology in the recent past. The analysis concerns the policies for controlling tuberculosis in Bulgaria in the decade after 1944, when the BCG vaccine introduction programs were deployed in the country, placed in the context of the political tension between East and West in international health policies.^5^ Stepping into historiographical discussions and unpublished Bulgarian archives, several questions are addressed: what were the ideological imprints of the ‘Bulgarian’ BCG strain’s history in the context of international health policies in the Cold War era? How decisive was the influence of the USSR in health policy in Bulgaria after 1944? Was there a ‘Bulgarian BCG’ in this period, or is this a legend born in the Cold War of strains?

Tuberculosis is a disease that not only had a significant impact on the development of general mortality and life expectancy but also played a significant role in the so-called *epidemiologic transition –* the fundamental change in the historical landscape of the causes of morbidity and mortality.^6^ Until the 1960s, it was tuberculosis that concentrated the effects of real suffering and large-scale health and social policies in many countries. For more than a century, vaccinations had evolved from their once modest role in public health to become a cornerstone of global health policies in the second half of the 20th century.^7^ The introduction of state and global policies of *Mycobacterium bovis* bacillus Calmette-Guérin (BCG) vaccination in the 1950s turned it into a kind of *technological fix.* ^8^ In this period, vaccination policies were also charged with political significance, making BCG an important symbol of political rivalry in the Cold War.^9^ This is the context in which the historical case of the ‘Bulgarian’ BCG vaccine is considered.

BCG is the only live attenuated vaccine available against tuberculosis. The BCG vaccine sub-strains, which are currently in use, are all progenies of the original strain attenuated by Calmette and Guérin during 1908–1921.^10^ The BCG vaccine strains used worldwide represent a family of daughter sub-strains with distinct genotypic characteristics. Subsequent to its first use on humans in France in 1921, daughter strains were distributed around the world for use in vaccine manufacture. As a result of repeated passage under different conditions in different laboratories worldwide, the BCG vaccine strains diverged genetically until lyophilisation was introduced in the 1960s to store freeze-dried seed lots for vaccine production.^11^ The existence of different BCG (sub)strains began to be described in the 1940s – precisely when BCG vaccination became important for tuberculosis elimination programs in many countries. Variation in techniques for culturing and storage between laboratories has resulted in genetically divergent strains of the original BCG vaccine that are currently being employed. Currently, the most used strains in the world are *Pasteur 1173 P2*, Danish (*Copenhagen–1331, Glaxo 1077* – derived from the Danish strain), Japanese (*Tokyo–172-1*), Russian (*BCG-I*, *Moscow–368*), Brazilian (*Moreau/RDJ*), and Bulgarian (*SL222 Sofia*).^12^ Although several strains of BCG are currently in use, there is no agreement around one strain being superior to the others, even though different strains of BCG vary considerably in bacterial viability, which may correlate with immunostimulatory capacity.^13^

## BCG in global health policies after the Second World War

Organised efforts to prevent the spread of infectious diseases, particularly tuberculosis, through mass vaccination, began after the First World War, but progressed relatively slowly in the interwar period.^14^

Central and South-Eastern Europe, together with the Soviet Union, functioned as a kind of “frontier zone” of European health care in the interwar period.^15^ In this region, the League of Nations Health Organization (LNHO) and the Rockefeller Foundation concentrated on epidemiological cooperation and the development of social medicine. These initiatives integrated the Soviet Union, as well as Central and South-Eastern European countries, into emerging global public health networks and the broader debates on “global health”.^16^

The creation of the Soviet Union in the 1920s generated a radically new model of public health care based on the socioeconomic determinants of disease, prevention, and universal access, governed by comprehensive state control – the ‘Semashko’ healthcare system.^17^ The Soviet Communist discourse on the management of health as a biological and social phenomenon led to the unprecedented planning and centralisation of medical care.^18^ In the mid-1930s, the People’s Commissariat of Public Health (hereafter Narkomzdrav) introduced compulsory smallpox vaccination, a nationwide plan to combat malaria, and by 1938 it had expanded tuberculosis vaccination of newborn children to sixty-five cities and industrial centres.^19^ In general, state-socialist approaches to health care combined pre-1945 traditions that emphasised the socioeconomic roots of disease with the introduction of centralised institutions that rested on authoritarian state rule. Free access to health care merged with intervention and control: This was the medical version of ‘socialist modernism’,^20^ which the communist regimes opposed to the practices of the rival ‘capitalist camp’.^21^

In the late 1940s, tuberculosis treatment was on the verge of a so-called antibiotic revolution.^22^ At the same time, the international community of experts involved in anti-tuberculosis policies was consensual regarding the effectiveness and reliability of the BCG vaccine.^23^ The more common ways of controlling the disease, until that time, through diagnostic chest X-rays and systematic isolation of patients were almost impossible in the early years of post-war Europe. This situation necessitated international initiatives to introduce mass immunisation campaigns as a means of controlling the spread of tuberculosis.^24^

International campaigns found their focus in the joint program for mass vaccination against tuberculosis, established in 1946 by the *United Nations International Children’s Emergency Fund* (UNICEF).^25^ With the establishment of the *World Health Organization* (WHO), it became the main coordinator of international health control programs, including the one for combating tuberculosis (in addition to those for venereal diseases, malaria, polio, smallpox, etc.).

In May 1947 the *Danish Red Cross* began a pilot program in Poland to test children for tuberculosis, immunise those who test negative with the BCG vaccine, and train local doctors, nurses, and medical students in diagnosis and vaccination. In the first six months of the campaign, 46,000 were vaccinated.^26^ In 1948 – the year the First International BCG Congress was held, in Paris^27^ – Norwegian and Swedish relief organisations agreed to work with the Danes, creating what became famous as the **Joint Enterprise*,* where UNICEF provided financial support. On 1 July 1948, the Joint Enterprise, later renamed *International Tuberculosis Campaign* (ITC), officially took over the activities started by the *Danish Red Cross.* One of the first actions of the newly established WHO was the creation of the *Committee on Biological Standardization* – to ensure that the BCG vaccine produced by various laboratories around the world was of standard quality and strength.^28^ It was planned that the ITC would operate until 30 June 1951, after which the campaign would be integrated into the WHO work program as a priority activity. Institutional division of labour was also agreed, where UNICEF would be responsible for the supply of the BCG vaccine and individual governments would be responsible for managing the program in their territories.^29^ Under these circumstances, BCG vaccination achieved its status as the preferred means of controlling tuberculosis. Meanwhile, research in the Nordic countries had contributed to changing the understanding and use of BCG vaccination. Perhaps the most important factor in determining health policy in this area, however, was the modest cost of BCG vaccination compared to other treatments.^30^

By the end of the 1940s and the beginning of the 1950s, the differences in the visions of the East and the West countries about how public health systems should be created and organised became clear. The Soviet Union’s withdrawal from the WHO in 1949^31^ also meant the withdrawal of the countries with pro-Soviet regimes from the organisation. In the entire Eastern Bloc, including Bulgaria in 1951, the Soviet health system ‘Semashko’ was finally established. In the same year, mandatory BCG vaccination was also introduced in most of these countries.^32^

As a result of the (self-)sovietisation and (self-)isolation, the latest research on the development of new vaccines, and especially research on the problems associated with the mass production of vaccines, became less accessible to Soviet and Eastern European specialists. In these contexts, the BCG vaccine also became a terrain of political rivalry, and campaigns for its administration were given enormous ideological importance.^33^ A particular focus in the political vaccine race was the development and production of a reliable freeze-dried BCG vaccine.

## The ideological ‘freezing’: the freeze-dried (lyophilised) BCG vaccine

The strain BCG was brought from the Pasteur Institute in Paris to the State Institute of Standardization and Control of Medical and Biological Products in Moscow in 1925 by Dr. Lev A. Tarasevich,^34^ who named it *BCG-1.* In the 1930s, the main challenge was the storage of the vaccine: the problem was that liquid BCG had a shelf life of 15 days, after which the number of live bacteria in it dramatically decreased. That is why, since the end of the 1930s, the possibility of creating a ‘dry’ BCG vaccine was increasingly discussed. By freeze-drying the vaccines, the treatment could safely be stored, transported over long distances, and diluted when needed.^35^ Already in the late 1930s, several Soviet scientists were working on the possibilities of lyophilisation of the BCG vaccine. The experimental breakthrough occurred in 1941, when Е. N. Leshchinskaya and А.М. Vakengut, working at the BCG laboratory of the Central Institute of Experimental Medicine, published a description of their technology^36^ for the preparation of a lyophilised BCG vaccine with a 12-month shelf life.^37^

Immediately after the end of the Second World War, at the Pasteur Institute in Paris and in other world-leading laboratories, work was carried out in the direction of improving the possibilities of producing a lyophilised vaccine. In February 1946, for example, the *American Review of Soviet Medicine* published an editorial article on ‘The Immunizing Value of the BCG Dry Glucose Vaccine’. It presented the results of Leshchinskaya and Vakengut’s experiments and the possibilities that the mass production of dry BCG vaccine could give, centralizing the production and increasing the range of the vaccines.^38^ In 1947, it was precisely the Pasteur Institute that undertook the study of the problem and the coordination of the freeze-dried BCG vaccine production for Europe. The Parisian technique stepped on the publications of Leshchinskaya and Vakengut in the specialised journal,^39^ but gradually expanded the transfer of ideas and practices^40^ – a transfer from which the Eastern Bloc effectively distanced itself almost until the end of the 1950s.

## BCG policies in Soviet Bulgaria: the ‘cold war’ of strains

‘The fight against tuberculosis in our country is built on the principles of socialist health care, based on the example and rich experience of Soviet health care’.^41^

The Bulgarian experience with BCG begins with the activities of Prof. Toshko Petrov.^42^ In 1926, he brought the strain from the Pasteur Institute and organised its cultivation in the modest conditions of the laboratories of the Sofia Bacteriological Institute.^43^ By 1928, Prof. Petrov and his team managed to administer the vaccine to only 371 children, and in 1931 he published the results of the vaccination of 1,170 newborns. After T. Petrov died in 1942, his associates Prof. L. Tsvetkov and Prof. B. Slavkov continued efforts to expand the scope of BCG vaccination. By the autumn of 1944, a total of about 7,000 children had been vaccinated.^44^

Although there were no military actions on the territory of Bulgaria during the Second World War, the country was in a state of martial law, and international relations were severed. In the winter of 1944, the building stock of the medical facilities in the capital was partially destroyed, the headquarters of many central institutions were evacuated to the countryside due to the aerial bombardment, and medical personnel was mobilised.^45^

With the political coup, carried out under conditions of Soviet occupation on 9 September 1944, the Communist Party-dominated coalition Fatherland Front came to power. This was the beginning of radical changes not only in the public health administration. The ‘breakthrough’ in ideological restructuring and the gradual establishment of the Soviet-style ‘socialist organisation’ of health care was drafted.^46^

At the end of 1947, UNICEF made an official proposal to the Bulgarian government and the teams of the Danish Red Cross to begin research and introduce a larger scale BCG vaccination – they had already been working in six European countries.^47^ By 1948, the Bulgarian government almost accepted this option of a ‘*gradually organized and well-controlled application*’ of the BCG vaccine in Bulgaria.^48^

The parameters of the Bulgarian government’s agreement with UNICEF and the Danish Red Cross were finalised in June 1948. It was planned that three Danish teams of one doctor, one nurse, and one secretary with their cars would be admitted to the country to start the vaccinations and establish the application system. In addition, it was agreed that three Bulgarian doctors would be sent to Copenhagen to study the Danish experience in anti-tuberculosis vaccine production, taking into consideration its future production in Bulgarian laboratories.^49^ The problem was that after the establishment of the *Cominform*,^50^ the rapid implementation of such international initiatives became more difficult for the countries of the Soviet sphere of influence. At the end of July of the same year, a negative reaction came from the Soviet Union to the Bulgarian Ministry of Health: ‘vaccine manufactured in Copenhagen cannot be preferred in vaccination’.^51^ The Bulgarian government had to abandon the agreement, although it was already prepared and advertised in the press. Despite refusing to introduce mass vaccination according to the Danish method, the Bulgarian government accepted the Danish Red Cross’s proposal for support in the development of a locally produced BCG vaccine. For this purpose, in August 1948, the Ministry of Health sent a Bulgarian team on a scientific business trip to Copenhagen – Dr. M. Mondeshki (director of the Clinic of Phthisiology), Har. Boyadzhiev (director of the Department for the fight against tuberculosis in the Ministry), Dr. M. Zaharieva (head of the Central Institute for the Production of Serums and Vaccines), and doctors As. Tashkov and L. Nedev.^52^

In 1949, health experts from Moscow arrived in Bulgaria to pass on the Soviet experience and to prepare the implementation of BCG ‘on a larger scale and in a completely planned way’.^53^ The Ministry of Health organised immunisation with a BCG vaccine of its own production from the Soviet strain in the Fall of 1949 in the larger cities.^54^ In 1951, immunisation against tuberculosis in Bulgaria became mandatory for all newborns and children under the age of 18.^55^ By the end of 1952, about 300,000 children had been vaccinated.^56^

Also in 1949, as part of the Soviet bloc, Bulgaria left the WHO, and the country’s admission to the UN was delayed until 1955.^57^ At the same time, the Bulgarian government also decided to terminate the country’s membership in 8 international organisations^58^, including the International Union Against Tuberculosis.^59^ International contacts in the fight against tuberculosis were officially ‘frozen’ and existed until the end of the 1950s only within the socialist bloc.^60^

The establishment of the *National Research Institute of Epidemiology and Microbiology* (NIEM) in Sofia in 1950 also shows the mechanisms of imposing the radical ideological change in this sphere – the laboratories were given plans drawn up on the Soviet model for implementation, and the produced serums and preparations had to ‘fully meet the latest Soviet requirements’.^61^ One of the important tasks set before NIEM specialists in 1951 was ‘the restructuring of scientific research and pedagogical activity on the basis of the progressive teaching of Pavlov, Michurin, Lysenko’.^62^ Soviet specialists came to the institute as advisers, and several Bulgarian experts were sent to specialise in the Soviet Union – at the ‘Nikolay Fedorovich Gamaleya’ Institute of Epidemiology and Microbiology and the ‘Ilya Mechnikov’ scientific research institute in Moscow. Since the mid-1950s, NIEM had been working with ‘32 original Soviet instructions’,^63^ developed by the ‘Tarasevich’ State Control Institute for Serums and Vaccines, that contained specific (medical) instructions with a described methodology for preparation, storage, and shelf life, control, and application of the various vaccines.^64^ A confidential report prepared by the Ministry of Health and sent on 13 March 1951 to the chairman of the Soviet-Bulgarian commission for scientific and technical cooperation briefly presented NIEM’s work after 1944:

In its work in the past, this institute has received help from some Western European institutes. So, for example production and diagnostic strains and standard preparations were obtained from the Frankfurt Institute in Germany. After September 9, 1944, our institute did not maintain contact with the institutes in capitalist countries, although they sought such. Only some standard preparations from the Copenhagen institute are obtained, but not systematically, and free of charge… Since 1949, Soviet methods have been introduced in the work of our institute with the help of Soviet specialists. Work is conducted with Soviet strains and standard preparations.^65^

## The ‘Srebra Rodopska’ case and the Bulgarian version of the BCG vaccine

The popular 2020 media version of Dr. Srebra Rodopska’s biography can be retold like this: in 1948, she was sent by the Bulgarian government for specialisation to Paris, where she worked in the laboratories of the Pasteur Institute, and on her return to Bulgaria ‘semi-legally’ brought BCG to Sofia. Here, she ‘improved’ the French strain with the Soviet strain and eventually developed the best version – the ‘Bulgarian’ BCG. Even an expert version presented at an academic forum in 2008 gives a similar interpretation:

In 1948, Dr. Srebra Rodopska brought the BCG strain from Paris after having been acquainted with the production of liquid BCG vaccine in the Pasteur BCG Laboratory. As in about 1% of the newborns vaccinated orally suppurative cervical lymphadenitis (inflammation of the lymph nodes in the neck) had appeared, the Paris strain was substituted with the Moscow strain BCGI. After the new strain was introduced for production in Bulgaria, cervical lymphadenitis (inflammation of the lymph nodes in the neck) became an extremely rare complication.^66^

The analysis of the documentary traces, especially those about the activities of the *Research Institute of Tuberculosis* , stored in the Central State Archive, gives greater clarity specifically about Dr. Rodopska and the institutional contexts of her work. After graduating from the Faculty of Medicine of Sofia University in 1941, she began working as a bacteriologist in the Research Institute of Epidemiology and Microbiology in Sofia.^67^ Immediately after the coup of 9 September 1944, Srebra Rodopska became a member of the Bulgarian Workers’ Party (communists), and by the spring of 1945, she was already ‘Head of Department’ at the institute.^68^

An important element in the biography of Srebra Rodopska is that her husband, Dr. Tasho Tashev (later a professor and academician, specialist in gastroenterology and dietetics), became the personal physician of the Bulgarian communist leader Georgi Dimitrov upon his return from the Soviet Union in 1945.^69^ The documents in Prof. Tashev’s professional file from the archival holding of the Medical Academy in Sofia show that in May 1947, by special decree of the Council of Ministers, Dr. Tashev was sent on a ‘one-year scientific business trip’ to Paris.^70^ His wife, Dr. Srebra Rodopska, also left for the French capital with him. In the archive of the Council of Ministers of the People’s Republic of Bulgaria, a folder labelled ‘Personal file of Srebra Rodopska’ is preserved. According to the documents in the case, she was also sent on a ‘scientific business trip’ to Paris (1 September 1947–30 May 1948) by special government decision – as an ‘employee’ of the Council of Ministers.^71^ Such high-level decisions were not common and the fact is telling about the authorities’ special attitude to the family of the former doctor of the leader of the Bulgarian Communist Party. Two more foreign business trips of Dr. Rodopska are noted – in Copenhagen (31 May 1948–20 June 1948) with a group of doctor-bacteriologists, and in Moscow in 1949 together with her husband.^72^

Srebra Rodopska’s secondment to the Pasteur Institute was important because of the opportunity to update herself on the Institute’s work in developing an effective lyophilised BCG (freeze-dried; preserved by dehydration under vacuum). With the (self-)imposed isolation of Soviet and Eastern European bacteriologists from their international colleagues, Dr. Rodopska was among the few Eastern European experts who were allowed to travel and who had access to current discussions in the field beyond the Iron Curtain. After returning to Sofia, she was sought after as the main expert on these matters. As early as 1950, the application of freeze-dried BCG in Bulgaria was discussed in the Department for the Fight Against Tuberculosis at the Ministry of Public Health:

On the matter of the dry vaccine, Dr. Rodopska sent a special letter in which it was said that currently there are no conditions for the preparation of a dry vaccine and that it is necessary for a person to specialize in the Soviet Union, as different methods and different results are reported there… What Dr. Rodopska wants, we will try to fulfill… More detailed instructions can be requested through the Comrade Minister of the Soviet Union.^73^

A year later, as head of the BCG Department at NIEM, Srebra Rodopska prepared a ‘confidential’ report on the possibilities for production and mass application of the BCG vaccine in Bulgaria. From it, it is clear that work was being done only on Soviet instructions and with the Soviet sub-strain of BCG:

The production of the BCG vaccine for mass usage began on 19.10.1949 – at the insistence of the Soviet instructors and according to Soviet instruction that was passed on to us by them… Currently, the vaccine is produced according to the latest Soviet instruction brought by Comrade Minister Petar Kolarov, approved by the Soviet Ministry of Health on 25.04.1950. According to the latest instruction, the production laboratory must annually replace the BCG strain with a new one obtained from the Central Control Scientific Institute ‘Tarasevich’ in Moscow.^74^

At the end of 1954, the *technology for the production of the dry anti-tuberculosis BCG vaccine was officially approved.* ^75^ However, its production in Bulgaria was terminated almost immediately: due to contamination of the sterile environment in the institute’s laboratories in December 1954, guinea pigs injected with experimental cultures from the production strain BCZ I-104 (received from the USSR in 1952) and from strain BCG I-117k (received from the USSR in 1953) were compromised.^76^ For this reason, Srebra Rodopska was sent to the Soviet Union to receive new instructions and protocols. BCG production was resumed under the ‘new Soviet instruction’ with a strain she brought from Moscow: a ‘pure’ line of BCG-117K.^77^ The reports on the work of the Institute for State Control of Medicinal Products in Sofia showed that in the following years, for the preparation and control of the BCG vaccine, work continued entirely according to the Soviet instructions prepared by Prof. Vygodchikov.^78^ In 1957, work continued ‘only with the BCG strain, which was stored, tested, and distributed by the “Tarasevich” Institute. Obtaining the strain for the production of the vaccine from other institutes is prohibited’.^79^

Regular production of lyophilised vaccines in Bulgaria began in 1963. Only in 1972, was *BCG SL222 Sofia* standardised: a seed lot sub-strain descending from the Russian *BCG-I* (seed lot 374a) strain was introduced.^80^ The strain was received at the National Center of Infectious and Parasitic Diseases for the production of the freeze-dried live BCG vaccine. Primary seed lot (Master Seed) SL222 Sofia of the Bulgarian BCG vaccine was produced in May 1972 directly from generation 374a of the Russian strain BCG-I.^81^

## Conclusion

Through the 1950s, Eastern and Western bloc countries had different visions of how public health systems should be established and organised. Political leaders on both sides of the ideological divide were convinced that demonstrating success in controlling the spread of infectious diseases would reflect their respective political systems. For politicians, vaccines had become symbols of ideological rivalry, their application loaded with political significance.^82^ The coup of 9 September 1944 in Bulgaria, marked the beginning of radical changes not only in the public health administration. The ‘breakthrough’ in the ideological restructuring and the gradual establishment of the ‘socialist organisation’ of Soviet-type health care was planned. In 1951, the institutional transformation of public health care on the model of the Soviet system was completed.^83^

Vaccine production in communist Bulgaria was significantly determined by external political and economic factors. One possible internal impetus could be the revolutionary goal of the new regime to reduce morbidity and mortality from infectious diseases, which were on the rise in the immediate post-war context. In addition to obvious health-related reasons, ideological motives also underpinned efforts to improve the general health of the population. Namely, in the early post-war period it was important for the socialist regime to legitimise itself by demonstrating the superiority of the new system over the previous one. Intensified and improved vaccine production served to demonstrate the rapid social development and modernisation taking place in the established socialist order. Throughout the period up to 1989, BCG campaigns in Bulgaria closely followed the Soviet model in the production and standards for the preparation of the vaccine itself, as well as the organisation of mandatory immunisations from 1951 onwards.

The re-emergence of Dr. Srebra Rodopska in the Bulgarian public sphere during the COVID-19 pandemic can be read as part of a broader turn towards symbolic re-nationalisation of scientific authority. Amid global uncertainty, many states sought reassurance through narratives of national medical achievements. In Bulgaria, Rodopska’s story filled a symbolic gap: a female scientific pioneer, ‘discoverer’ of a vaccine that now allegedly protected the nation. This constructed memory aligned well with contemporary anxieties and aspirations, elevating her to the status of a ‘public health hero’. While not driven by political elites per se, this discursive framing served a populist logic, transforming historical science into a usable past and a national claim to biomedical exceptionality.

By the late 1950s, debates over vaccine efficacy and safety had become the stage for propaganda wars in which sometimes ‘science and politics fused into one’. Vaccines became an integral part of post-war biopolitics, reflecting political, economic, and ideological commitments in medicine and science. The case of the ‘Bulgarian’ BCG vaccine, which turns out to be ‘Soviet’, exemplified how vaccines were instrumentalised in the ongoing propaganda war. The introduction and making of the legendary ‘Srebra Rodopska’ case in 2020–2021 is an example of how Cold War-era vaccine nationalism can be reactivated and transformed into forms of pandemic-era populism, drawing on past medical authority to produce new national myths.

## Coda: Bulgaria’s divergence in the Cold War vaccine landscape

While countries like Poland, Czechoslovakia, and Hungary permitted the Danish Red Cross to conduct BCG vaccination programs, Bulgaria rejected direct cooperation after initially preparing to accept it. This divergence is striking. What makes Bulgaria a special case may lie in the timing and intensity of its early Soviet alignment. The Bulgarian government’s abrupt reversal of agreements with the Danes in 1948 coincides with Stalin’s growing insistence on ideological conformity and Bulgaria’s close political alignment with the USSR at the time. While other Eastern Bloc countries allowed limited Western cooperation in health programs, Bulgaria’s self-isolation appears more severe, possibly as a performance of loyalty. This makes Bulgaria a compelling case for future comparative research. Why were some countries granted more latitude in negotiating between East and West? What institutional, political, or personal dynamics determined the thresholds of permissible foreign involvement? Exploring such questions would benefit from archival work in the UNICEF and Danish Red Cross collections and offer a richer understanding of how Cold War geopolitics shaped health policy on the periphery.

## Footnotes

1 Hristo Golemanov, *Metodichni Materiali po Protivotuberkulozna Propaganda* (Sofia: Nauka i izkustvo, 1952), 98.

2 See Jakub Kulesza et al., ‘BCG and SARS-CoV-2—What Have We Learned?’, Vaccines, 10, 10 (2022), 1641, 10.3390/vaccines10101641 36298506 PMC9610589; Aaron Miller et al., ‘Correlation between Universal BCG Vaccination Policy and Reduced Morbidity and Mortality for COVID-19: An Epidemiological Study’, *medRxiv*, 14 (2020), https://doi.org/10.1101/2020.03.24.20042937; Hongseok Namkoong , Nobuyuki Horita , and Risa Ebina-Shibuya , ‘Concern over a COVID-19-Related BCG Shortage’, International Journal of Tuberculosis and Lung Disease, 24, 6 (2020), 642–643, 10.5588/ijtld.20.0240 32552997; Konstantin Chumakov et al., ‘Old Vaccines for New Infections: Exploiting Innate Immunity to Control COVID-19 and Prevent Future Pandemics’, Proceedings of the National Academy of Sciences of the United States of America, 118, 21 (2021), 10.1073/pnas.2101718118 PMC816616634006644.

3 They analysed whether the spread of the coronavirus changes according to the Cold War-era border between East and West Germany, whose vaccination policies differed before their 1990 reunification. Maxim Pinkovskiy and Richard Bluhm , ‘The Spread of COVID-19 and the BCG Vaccine: A Natural Experiment in Reunified Germany’, The Econometrics Journal, 24, 3 (2021), 353–376, 10.1093/ectj/utab006 .

4 More about the media coverage of Srebra Rhodopska’s image in Bulgaria: see Aneliya Kasabova , ‘Kak Pandemiyata COVID-19 Stimulira Interdistsiplinarni Nauchni Izsledvaniya’, in Vihra Baeva and Angelina Ilieva (eds.), Etnologiya i epidemii: Sotsiokulturni izmereniya na pandemiyata ot COVID-19 (Sofiya: Izdatelstvo na BAN, 2021), 39–50 .

5 For similar studies in these contexts regarding tuberculosis, see Donna Harsch , ‘Medicalized Social Hygiene? Tuberculosis Policy in the German Democratic Republic’, Bulletin of the History of Medicine, 86, 3 (2012), 394–423 23241911 10.1353/bhm.2012.0059; Šárka Caitlín Rábová , ‘Between Public Health and Propaganda: Tuberculosis in Czechoslovakia in the First Decades of the Communist Regime’, The Hungarian Historical Review: New Series of Acta Historica Academiae Scientiarum Hungaricae, 13, 3 (2023), 433–90.

6 See Abdel R. Omran , ‘The Epidemiologic Transition. A Theory of the Epidemiology of Population Change’, Milbank Memorial Fund Quarterly, 49, 1 (1971), 509–385155251 ; Johan P. Mackenbach , A History of Population Health. Rise and Fall of Disease in Europe (Leiden: Brill, 2020); Alexander Mercer , Infections, Chronic Disease, and the Epidemiological Transition: A New Perspective (Rochester, NY: University of Rochester Press, 2014).

7

Stuart Blume , ‘The politics of global vaccination policies’, in Colin McInnes , Kelley Lee , and Jeremy Youde (eds.), The Oxford Handbook of Global Health Politics, *2020* : online edn (New York: Oxford Iniversity Press), 10.1093/oxfordhb/9780190456818.001.0001 , accessed 26 Mar. 2024), 599. Vaccines had a prominent role in the early 20th Century (before the invention of antibiotics), then were sidelined from the 1940s but never totally modest and made a return in the later 20th Century. See, for example, Scot Podolsky , Pneumonia before Antibiotics: Therapeutic Evolution and Evaluation in Twentieth-Century America (Baltimore: Johns Hopkins University Press, 2006).

8 Daniel Sarewitz and Richard Nelson, ‘Three Rules for Technological Fixes’, *Nature*, 456, (2008), 871–2, https://doi.org/10.1038/456871a; Nicholas B. King, ‘Technological fixes and antimicrobial resistance’, in Euzebiusz Jamrozik and Michael Selgelid Jamrozik (eds.), *Ethics and Drug Resistance: Collective Responsibility for Global Public Health*, Public Health Ethics Analysis, 5 (Springer: Cham, 2020), https://doi.org/10.1007/978-3-030-27874-8_19.

9 See Socrates Litsios , ‘The Long and Difficult Road to Alma-Ata: A Personal Reflection’, International Journal of Health Services, 32, 4 (2002), 709–32, 10.2190/RP8C-L5UB-4RAF-NRH2 12456122 .

10

Todia P. Setiabudiawan , et al., ‘Protection against Tuberculosis by Bacillus Calmette-Guérin (BCG) Vaccination: A Historical Perspective’, Med, 3, 1 (2022), 6–24 35590145 10.1016/j.medj.2021.11.006; Samreen Fatima , et al., ‘Tuberculosis Vaccine: A Journey from BCG to Present’, Life Sciences, 252 (2020), 11759410.1016/j.lfs.2020.11759432305522; Stefan Panaiotov , et al., ‘Complete Genome Sequence, Genome Stability and Phylogeny of the Vaccine Strain Mycobacterium bovis BCG SL222 Sofia’, Vaccines, 9, 3 (2021), 237, 10.3390/vaccines9030237 PMC800055833803448; Luca Simona and Traian Mihaescu , ‘History of BCG Vaccine’, Maedica, 8, 1 (2013), 53–824023600 PMC3749764; Christian Bonah , ‘The “Experimental Stable” of the BCG Vaccine: Safety, Efficacy, Proof, and Standards, 1921–1933’, Studies in History and Philosophy of Science Part C: Studies in History and Philosophy of Biological and Biomedical Sciences, 36, 4 (2005), 696–721 10.1016/j.shpsc.2005.09.00316337557.

11 For example, BCG-Moreau and BCG-Japan, two strains obtained from the Pasteur Institute before 1926; strains obtained after 1931 from the Pasteur institute, such as BCG-Denmark, BCG-Tice, and BCG-Glaxo. See Abdallah M. Abdallah and Marcel A. Behr , ‘Evolution and strain variation in BCG’, in Sebastien Gagneux (ed.), Strain Variation in the Mycobacterium Tuberculosis Complex: Its Role in Biology, Epidemiology and Control (Cham: Springer, 2017), 155–69, 10.1007/978-3-319-64371-7_8.29116634

12

Tania Cernuschi , et al., ‘Bacillus Calmette-Guérin (BCG) Vaccine: A Global Assessment of Demand and Supply Balance’, Vaccine, 36, 4 (2018), 10.1016/j.vaccine.2017.12.010 PMC577763929254839; Tzvetelina Stefanova , ‘Quality Control and Safety Assessment of BCG Vaccines in the Post-genomic Era’, Biotechnology & Biotechnological Equipment, 28, 3 (2014), 387–9126019525 10.1080/13102818.2014.927200PMC4433890.

13 Setiabudiawan, et al., *op. cit.* (note 10).

14

Josep L. Barona , Health Policies in Interwar Europe: A Transnational Perspective (London and New York: Routledge, 2018); Paul Weindling , ‘Public Health and Political Stabilization: The Rockefeller Foundation in Central and Eastern Europe between the Two World Wars’, Minerva, 31, 3 (1993), 253–6711623202 10.1007/BF01098623; Simona and Mihaescu, *op. cit.* (note 10); Todia P Setiabudiawan, *et al*., *op. cit.* (note 10). For the Bulgarian case, see Milena Angelova , Sotsialnata bolest. Tuberkulozata v Balgariya prez parvata polovina na ХХ vek (Blagoevgrad: UI Neofit Rilski, 2021).

15

Derek Aldcroft , Europe’s Third World: The European Periphery in the Interwar Years (Aldershot: Ashgate, 2006).

16

Iris Borowy , *Coming to Terms with World Health* . The League of Nations Health Organisation 1921–1946 (Berlin: Peter Lang, 2009), 85–94 ; Simon Peng-Keller , Fabian Winiger , and Raphael Rauch , The Spirit of Global Health: The World Health Organization and the ‘Spiritual Dimension’ of Health, 1946–2021 (Oxford University Press, 2022), 32 ; Weindling, *op. cit.* (note 14).

17 For recent work on the Soviet model of public health (the ‘Semashko’ system), see Andreas Heinrich , ‘The emergence of the socialist healthcare model after the First World War’, in Frank Nullmeier , Delia González de Reufels , and Herbert Obinger (eds.), International Impacts on Social Policy. Global Dynamics of Social Policy (Cham: Palgrave Macmillan, 2022), 35–46 ; Norman Naimark , ‘The Sovietization of East Central Europe 1945–1989’, in Norman Naimark , Silvio Pons , and Sophie Quinn-Judge (eds.), The Cambridge History of Communism. Vol. II: The Socialist Camp and World Power 1941–1960s (Cambridge: Cambridge University Press, 2017), 63–86 ; Susan G Solomon and John F. Hutchinson (eds.), Health and Society in Revolutionary Russia (Bloomington: Indiana University Press, 1990).

18 See Marius Turda , ‘History of medicine in Eastern Europe, including Russia’, in Mark Jackson (ed.), Oxford Handbook of the History of Medicine (Oxford: Oxford University Press, 2011), 216 ; Charles Webster , ‘Medicine and the welfare state 1930–70’, in Roger Cooter and John Pickstone (eds.), Companion of Medicine in the Twentieth Century (London: Routledge, 2003), 125–40.

19

Michael David , ‘Vaccination against tuberculosis with BCG. A study of innovation of public health, 1925–41’, in Francis Bernstein , Christopher Burton , and Dan Healey (eds.), Soviet Medicine: Culture, Practice, and Science (DeKalb: Northern Illinois University Press, 2010), 132–54.

20

Dora Vargha , ‘The Socialist World in Global Polio Eradication’, Revue d’études comparatives Est-Ouest, 1, 1 (2018), 88 .

21

Bogdan C. Iacob , ‘Health’ in James Mark and Paul Betts (eds.), Socialism Goes Global: The Soviet Union and Eastern Europe in the Age of Decolonization (Oxford University Press, 2022), 255–89, 10.1093/oso/9780192848857.003.0008.

22

Scott H. Podolsky and Anne Kveim Lie , ‘Futures and their uses: antibiotics and therapeutic revolutions’, in Jeremy A. Greene , Flurin Condrau , and Elizabeth Siegel Watkins (eds.), Therapeutic Revolutions: Pharmaceuticals and Social Change in the Twentieth Century (University of Chicago Press, 2016), 10.7208/chicago/9780226390901.003.0002 ; Robert Bud , Penicillin: Triumph and Tragedy (Oxford: Oxford University Press, 2007). Regarding penicillin as a ‘powerful medical instrument, a commodity, a warfare tool, and a cultural and political symbol during the earliest phase of the Cold War’, and instrument of propaganda and political strategies, affecting pharmaceutical development both in the Western and the Eastern blocs, see Mauro Capocci , ‘Cold Drugs. Circulation, Production and Intelligence of Antibiotics in Post-WWII Years’, Medicina nei Secoli: Journal of History of Medicine and Medical Humanities, 26, 2 (2014), 401–2226054208.

23 Marcin Stasiak, ‘The Power of Habits: BCG Vaccination and Building the Socialist State in Post-War Poland’, *Social History of Medicine*, hkae036, (2024), https://doi.org/10.1093/shm/hkae036. For an overview of WHO policies for tuberculosis control, see Mario C. Raviglione and Antonio Pio , ‘Evolution of WHO Policies for Tuberculosis Control, 1948–2001’, The Lancet, 359 (2 March 2002), 775–8010.1016/s0140-6736(02)07880-711888605.

24 On the historiography of the beginnings of mass BCG vaccination in individual countries and the beginnings of global vaccination policies, see Linda Bryder , ‘“We Shall Not Find Salvation in Inoculation”: BCG Vaccination in Scandinavia, Britain and the USA, 1921–1960’, Social Science & Medicine, 49, 9 (1999), 1157–1167, 10.1016/S0277-9536(99)00157-4 10501638 ; George W. Comstock , ‘The International Tuberculosis Campaign: A Pioneering Venture in Mass Vaccination and Research’, Clinical Infectious Diseases, 19, 3 (1994), 528–40, 10.1093/clinids/19.3.528 7811874 ; Marina Gheorghiu , ‘Antituberculosis BCG Vaccine: Lessons from the Past’, in Stanley Plotkin and Bernardino Fantini (eds.), Vaccinia, Vaccination, Vaccinology: Jenner, Pasteur and Their Successors, Clinical Infectious Diseases (Paris; London: Elsevier, 1996), 87–94; Harsch, *op. cit.* (note 5); Michael Worboys and Flurin Condrau (eds.), Tuberculosis Then and Now: Perspectives on the History of an Infectious Disease (Ithaca, NY: McGill–Queen’s University Press, 2010); Elisabeth Dietrich-Daum , Die ‘Wiener Krankheit’. Eine Sozialgeschichte der Tuberkulose in Österreich (Wien: Verlag für Geschichte und Politik, 2007); Angelova, *op. cit.* (note 14).

25 Under this program from 1948 to 1955, 155 million children were tested and 60 million were vaccinated in different parts of the world – see Luca, *op. cit.* (note 10), 56.

26

George W. Comstock , ‘The International Tuberculosis Campaign: A Pioneering Venture in Mass Vaccination and Research’, Clinical Infectious Diseases, 19, 3 (1994), 528–397811874 10.1093/clinids/19.3.528.

27 In 1948 Guérin was chairman of the First International Congress on BCG.

28 Bonah, *op. cit.* (note 10).

29 Blume, *op. cit.*, (note 7), 586–7.

30

Robert Loddenkemper and John F. Murray (eds.), Tuberculosis and War. Lessons Learned from World War II (Karger Publishers, 2018), 220, 10.1159/isbn.978-3-318-06095-9.

31 In 1958, the Soviet Union and the satellite states rejoined the WHO.

32

Milena Angelova , ‘Vizionerstvo i zdrave: modelat “Semashko” i savetizatsiyata na obshtestvenoto zdraveopazvane v Balgariya (1944–1951)’, Balkanistic forum, 30, 3 (2021), 74–103, 10.37708/bf.swu.v30i3.4 .

33 Litsios, *op. cit.* (note 9); Vargha, *op. cit.* (note 20); Blume, *op. cit.* (note 7).

34 Lev A. Tarasevich (1868–1927) was a Soviet microbiologist and pathologist. Tarasevich graduated from Novorossiia University in Odessa in 1891. From 1900 to 1902 he worked with I. I. Mechnikov in Paris. Between 1908 and 1924, he was a professor of the Advanced Courses for Women at the Second Moscow University. In 1918, Tarasevich founded in Moscow а centre for testing serums and vaccines (later the L. A. Tarasevich State Scientific Research Institute for the Standardization and Testing of Medicinal Biological Preparations). Tarasevich wrote works on vaccination against various infections and on the epidemiology and prophylaxis of tuberculosis. David, *op. cit.* (note 19), 132–54.

35

Henrice Altink , ‘“Fight TB with BCG”: Mass Vaccination Campaigns in the British Caribbean, 1951–6’, Medical History, 58 (2014), 475–9725284891 10.1017/mdh.2014.49PMC4176281.

36 By drying mycobacteria under high vacuum and at low temperature in a 33% glucose solution.

37

E.N. Leshchinskaya and A.M. Vakengut , ‘Usloviya dlitel’nogo sokhraneniya vaktsiny BTSZH’, Problemy tuberkuleza, 3 (1941), 63–6; YE.N. Leshchinskaya and A.M Vakengut , ‘Dal’neysheye izucheniye sukhoy glyukoznoy vaktsiny BTSZH’, Problemy tuberkuleza, 5–6 (1942), 47–50 (Е.Н., Лещинская and A.M. Вакенгут, ‘Условия длительного сохранения вакцины БЦЖ’, *Проблемы туберкулеза*, 3 (1941), 63–6; Е.Н. Лещинская, A.M Вакенгут, ‘Дальнейшее изучение сухой глюкозной вакцины БЦЖ’, *Проблемы туберкулеза*, 5–6 (1942), 47–50).

38 ‘Review of “The Immunizing Value of the BCG Dry Glucose Vaccine”’, *Public Health Reports (1896–1970)*, 62, 6, 12 (1947), 211–3, http://www.jstor.org/stable/4586007.

39

E.N. Leshchlnskaya , ‘The Immunizing Value of the BCG Dry Glucose Vaccine’, American Review of Soviet Medicine, 3 (1946), 210–521013600 .

40

R.E. Glover , ‘The Effects of (a) Freeze-Drying and (b) Low Temperature on the Viability of Mycobacterium tuberculosis’, Journal of Pathology and Bacteriology, 58 (1946), 111–421023169 10.1002/path.1700580119; J. Ungar , ‘Viability of Freeze-Dried BCG Cultures’, Tubercle, 30, 2–4 (1949), 10.1016/S0041-3879(49)80054-7 18102808 ; Sol Roy Rosenthal , ‘Conservation du Bacille de Calmette et Guerin (BCG) par Dessiccation apres Congelation’, Annales de l’Institut Pasteur, 75 (1948), 209–16;18098945; Frederik van Deinse , ‘Vaccination against Tuberculosis with Freeze-dried BCG Vaccine’, American Journal of Public Health, 41, 10 (1951), 1209–14. 10.2105/ajph.41.10.1209 14885513 PMC1525686 .

41

Todor Sharov , ‘Varhu organizatsiyata na borbata s tuberkulozata u nas’, in Hristo Golemanov (ed.), Metodichni materiali po protivotuberkulozna propaganda (Sofia: Nauka i izkustvo, 1952), 5–22 .

42 Toshko Petrov Toshkov (1872–1942) was a Bulgarian doctor and the founder of preventive medicine in Bulgaria. He established and directed the Institute of Hygiene in Sofia and the Department of Hygiene and Bacteriology at Sofia University’s Faculty of Medicine. He was the founder of the Microbiological Society, Society for Hygiene and Preventive Medicine.

43

St. Todorov, et al., Protivotuberkulozna vaktsinatsiya s BCG (Sofiya: Nauka i izkustvo, 1953), 18 .

44 The estimated incidence of tuberculosis in the country at that time was about 200,000, and the death rate was about 20,000 per year. Central State Archives (CSA), collection (coll.) 147Б, inventory (inv.) 3, archival unit (a.u.), 1773, sheets 1–2; Angelova, *op. cit.* (note 14), 126, 182, 318, 322.

45

Anelia Kasabova and Mincho Georgiev , ‘D-r Vladimir Kalaydzhiev ili vaplashtavaneto na modernostta’, Balkanistic Forum, 30, 3 (2021), 125–47, 127.

46 Following the Second World War, the epidemiological situation of tuberculosis was very serious throughout Europe, including in the countries of Eastern Europe. To cope with this situation, a centralised system for tuberculosis control centred on specialised institutions providing treatment and care services, as had been established in the Soviet Union before the war, was adopted in all Eastern European countries. With minor differences between countries, the tuberculosis control policies on prevention, diagnosis, and treatment followed the system in use in the Soviet Union. Many countries acquired experience in combating tuberculosis through applying this model. See Malgorzata Grzemska and Richard Zaleskis . ‘Tuberculosis control in the countries of Eastern Europe and the former Soviet Union’, in Mario C. Raviglione (ed.), Reichman and Hershfield’s Tuberculosis (CRC Press, 2006), 732 .

47 Czechoslovakia, Finland, Greece, Hungary, Poland, and Yugoslavia.

48 CSA, coll. 160, inv. 10, a.u. 345, sheet 135.

49 Angelova, *op. cit.* (note 14), 120.

50 Also known as Communist Information Bureau, Information Bureau of the Communist and Workers’ Parties, Informatsionnoye Byuro Kommunisticheskikh i Rabochikh Party (Russian). Cominform was the agency of international communism founded under Soviet auspices in September 1947, with nine members – the communist parties of the Soviet Union, Bulgaria, Czechoslovakia, Hungary, Poland, Romania, Yugoslavia, France, and Italy. Mounting tension, however, between Yugoslavia and the Soviet Union led ultimately to the expulsion of the Yugoslav communist party from the Cominform in June 1948. The Cominform’s activities consisted mainly of publishing propaganda to encourage international communist solidarity. The French and Italian parties were ineffective in carrying out the chief task assigned to them by the Cominform – to obstruct the implementation of the Marshall Plan and the Truman Doctrine. Cominform served more as a tool of Soviet policy than as an agent of international revolution. In 1956, as part of a Soviet program of reconciliation with Yugoslavia, the Soviet Union disbanded the Cominform. See Bojan Dimitrijevic , Cominform Crisis: Soviet-Yugoslav Stand-Off, 1948–1954 (Warwick: Helion Limited, 2023).

51 CSA, coll. 136, inv. 3, a.u. 70, sheet 18.

52 Angelova, *op. cit.* (note 14), 122.

53 Hristofor Mihaylov, *Borbata s tuberkulozata i organizatsiyata na protivotuberkuloznata pomosht prez perioda na vazstanovyavane narodnoto stopanstvo na NR Balgariya* (Sofia, 1966), 14.

54 CSA, coll. 136, inv. 13, a.u. 126, sheet 8.

55

T.B. Yablokova , et al., ‘Sravnitel’noye izucheniye sovetskoy i bolgarskoy vaktsin BTSZH’, Problemy tuberkuleza, 6 (1980), 60–47393916.

56 CSA, coll. 136, inv. 13, a.u. 126, sheet 9.

57

Paul Betts , ‘Rights’, in James Mark and Paul Betts (eds.), Socialism Goes Global: The Soviet Union and Eastern Europe in the Age of Decolonization (Oxford University Press, 2022), 180–220, 183, 10.1093/oso/9780192848857.003.0006 .

58 Some of the organisations from which the country left were The International Union Against Tuberculosis, The International Society for the Prevention of Blindness, The International Union Against Venereal Diseases, and The International Union Against Cancer – CSA, coll. 1481, inv. 1, a.u. 1054, sheet 15.

59
*Ibid.*, sheets 11–7.

60 Despite isolation from the international medical community by the late 1940s, during the critical moments of the Cold War, the bonds of Soviet medicine and public health care with the Western world were not fully broken and were strengthened during the de-Stalinisation that occurred during the second half of the 1950s, with a wide range of strategies and hopes to establish relationships with other countries, especially the USA. Thus, in 1957, the Soviet Union rejoined the World Health Organization (WHO), which it had left in 1949. See Miguel Marco Igual , ‘Vaccine Diplomacy during the Cold War (i), Heads or Tails. Heads: Mikhail Chumakov and the Oral Poliomyelitis Vaccine of Albert Sabin’, Neurosciences and History, 12, 2 (2024), 61–76 ; Paula A. Michaels , ‘Soviet Medical Internationalism amid Destalinization, 1953–1958’, The Soviet and Post-Soviet Review, 50, 1 (2022), 40–63 .

61 CSA, coll. 261, inv. 1, a.u. 1, sheet 30.

62 CSA, coll. 261, inv. 2, a.u. 1, sheet 14.

63 CSA, coll. 261, inv. 2, a.u. 13, sheet 17.

64 Kasabova *op. cit.* (note 43), 130–1.

65 CSA, coll. 375, inv. 5, a.u. 80, sheet 44.

66 M. Chouchkova, T. Stefanova, and A. Mihaylov, ‘Centenary of the BCG Vaccine’, *European Society of Mycobacteriology, 29th Annual Congress* (Plovdiv, 2008), 5–9.

67 CSA, coll. 136, inv. 18A, a.u. 745, sheets 1–3 – Council of Ministers, Personal file of Srebra Grozdanova Rodopska, an employee of the Council of Ministers (1948–1949), 1948.

68
*Ibid.*, 1.

69 Georgi Dimitrov (1882–1949) was a Bulgarian communist leader who became the post-Second World War prime minister of a communist-dominated Fatherland Front government in Bulgaria. Assuming dictatorial control of political affairs, he effected the communist consolidation of power that culminated in the formation of a Bulgarian People’s Republic in 1946. He served as General Secretary of the Bulgarian Communist Party from 1946 to 1949. From 1935 to 1943, he was the General Secretary of the Communist International. Marietta Stankova, *Georgi Dimitrov: A biography* (London; New York: I.B. Tauris, 2010).

70 Sofia State Archive, coll. 927, inv.7, a.u. 69, sheets 49, 69, 86 – Medical Academy, Service file of Prof. Dr. Tasho Tashev.

71 CSA, coll. 136, inv. 18A, a.u. 745, sheet 1.

72
*Ibid.*

73 CSA, coll. 160, inv. 11, a.u. 275, sheets 39–40 – Meeting of the Department of Tuberculosis at the Ministry of Public Health, 19 August 1950.

74 CSA, coll. 375, inv. 5, a.u. 80, sheet 46 – Confidential report by the head of the BCG department Dr. Srebra Rodopska of the Republican Institute of Epidemiology and Microbiology regarding the beginning of production and mass application of the BCG vaccine in Bulgaria, 04 May 1951.

75 CSA, coll. 471, inv. 1, a.u. 5, sheets 29–33, 62–7.

76 CSA, coll. 472, inv. 1, a.u. 5, sheets 27–8 **–** Report by Srebra Rodopska from 17 June 1955.

77 CSA, coll. 472, inv. 1, a.u. 5, sheets 25–6.

78 Grigoriy Vasilievich Vygodchikov (1899–1982) was a Soviet microbiologist; he was head of the State Institute of Standardization and Control of Biomedical Preparations named after. L. A. Tarasevich (until 1942), and later he also directed the Central Institute of Epidemiology and Microbiology within the Ministry of Health.

79 CSA, coll. 472, inv. 1, a.u. 5, sheets 1–10.

80 The *BCG-I* (seed lot 374a) lyophilised batch was produced in February 1971 in Moscow, and samples of it were sent to Bulgaria. The strain was received at the National Center of Infectious and Parasitic Diseases for the production of the freeze-dried live BCG vaccine. Primary seed lot (Master Seed) *SL222 Sofia* of the Bulgarian BCG vaccine was produced in May 1972 directly from generation 374a of the Russian strain *BCG-I* and has been used for vaccine production in Bulgaria. Panaiotov, *op. cit.* (note 10), 237.

81

A. Engibarov , M. Chouchkova , and C. Koychev , ‘Studies on the Quality Control of BCG Vaccine: Laboratory Examinations and Post-vaccination Control in the Field’, Developments in Biological Standardization, 58 (1986), 163–713596028 ; Olga Narvskaya , et al., ‘First Insight into the Whole-genome Sequence Variations in *Mycobacterium bovis* BCG-I (Russia) Vaccine Seed Lots and Their Progeny Clinical Isolates from Children with BCG-induced Adverse Events’, BMC Genomics, 21, 567 (2020). 10.1186/s12864-020-06973-5 32811436 PMC7437937 .

82

Anne-Marie Moulin , ‘The international network of the Pasteur Institute, scientific innovations and French tropisms’, in Charle, Christophe , Jürgen Schriewer , and Peter Wagner (eds.), The Emergence of Transnational Intellectual Networks and the Cultural Logic of Nations (Chicago: The University of Chicago Press, 2004), 135–62.

83 Angelova, *op. cit.* (note 14), 109.

